# The Effect of Intermittent Theta Burst Stimulation (iTBS) in Patients With Alcohol Use Disorder: Study Protocol for a Randomized Controlled Trial

**DOI:** 10.3389/fpsyt.2020.00210

**Published:** 2020-03-10

**Authors:** Chenxin Yuan, Hang Su, Tianzhen Chen, Valerie Voon, Jiang Du

**Affiliations:** ^1^ Shanghai Mental Health Center, Shanghai Jiao Tong University School of Medicine, Shanghai, China; ^2^ Department of Psychiatry, University of Cambridge, Cambridge, United Kingdom

**Keywords:** alcohol use disorder, iTBS, craving, randomized controlled trial, non-invasive brain stimulation

## Abstract

**Background:**

Repetitive transcranial magnetic stimulation (rTMS) is a non-invasive stimulation technique which has a treatment potential for alcohol use disorder. Intermittent theta burst stimulation (iTBS) is a new rTMS technique which is shorter in duration and thus with better tolerability and shows similar efficacy as rTMS for the treatment of depression. The effect of iTBS on reducing craving in alcohol use disorder patients requires further investigation.

**Methods:**

A randomized, controlled, single-blind, multicenter study with 60 alcohol use disorder patients randomized (2:1) to the iTBS group or the control group (sham iTBS). The stimulation target will be identical in the left dorsolateral prefrontal cortex (DLPFC). Baseline evaluations will be occurred before the intervention, after the intervention immediately, and 1 and 3 months after the intervention. The primary outcome of the study will be decrease of visual analogue scale (VAS) scores from baseline to the end of treatment.

**Discussion:**

This study is a randomized controlled trial to investigate the efficacy of left DLPFC iTBS in a population of alcohol use disorder patients, compared with sham iTBS. If it is effective for alcohol use disorder, it may provide a potential treatment which is tolerable, accessible, and clinical useful.

**Cinical Trial Registration:**

This study is registered in the ClinicalTrials with trial number NCT03932149. Registered 17 April 2019.

## Background

Alcohol dependence or alcohol use disorder (AUD) is a major international public health issue and a chronic disease with high relapse rates (1). Marked medical and psychiatric comorbidity and high numbers of premature deaths result from direct and indirect causes of excessive alcohol use (2). The disorder is associated with key societal impact goals influencing health related targets relevant to the Sustainable Development Goals (SDGs) of maternal and child health, mental health, injuries, and poisonings. Worldwide alcohol abuse was responsible for 3 million deaths (5.3% of all deaths) in 2016 and 132.6 million disability-adjusted life years (3). Critically however, the treatment of alcohol addiction is currently limited to acute withdrawal symptoms, but lacks effective interventions to reduce craving and prevent relapse, which are clinically the most relevant targets for the treatment of substance dependence. According to previous studies, non-conscious appetitive reactions occur when patients are exposed to alcohol-related cues and these reactions might explain why AUD patients relapse (4, 5). Furthermore, brain activity in reward-related areas in response to alcohol-related cues is positively related to the amount of post-relapse alcohol consumption (6). Thus, one way to effectively treat AUD may be through a targeted neurostimulation. An increasing number of studies have suggested a potential role for non-invasive neuromodulation as a means to target AUD (7, 8).

Repetitive transcranial magnetic stimulation (rTMS) is a non-invasive, cortical stimulation technique. An alternating electric current is applied from a coil which produces a magnetic field that depending on the frequency, can inhibit or activate cortical neurons both locally at the site of the coil and also have more distal network effects. The current results in high intensity magnetic pulses that pass through the skull and result in an electric current in the neural tissue, which can change cortical activity (7, 9). rTMS has been shown to be effective in large randomized controlled trial studies in a range of neuropsychiatric diseases including depression and obsessive-compulsive disorder (10, 11). With respect to disorders of addiction, a meta‐analysis revealed a significant anti‐craving effect of excitatory rTMS of the left dorsolateral prefrontal cortex (DLPFC) in patients with substance dependence (12). In AUD specifically, one study suggested that high-frequency rTMS on either right or left DLPFC significantly reduce craving scores in patients (13). In contrast, another study showed that a single rTMS stimulation session had no significant effects on alcohol craving in alcohol dependent patients emphasizing the potential need for repeated sessions (14). Several studies have reported the potential of rTMS to reduce craving in people with alcohol dependence, but multiple different parameters and protocols were reported with limited consistency (13–18).

Theta burst stimulation (TBS) is a rTMS technique that displays faster and more robust action compared to conventional rTMS protocols (19), and has excellent tolerability (20) and safety (21). intermittent theta burst stimulation (iTBS) and continuous theta burst stimulation (cTBS) are two different methods with facilitating and inhibitory effects, respectively (22). A randomized, multicenter, non-inferiority trial to compare iTBS with 10 Hz rTMS in patients with treatment-resistant depression targeting the left DLPFC demonstrated that iTBS was non-inferior to conventional 10 Hz rTMS in the treatment of depression (23). An increasing number of studies have focused on TBS in the field of addiction. One study demonstrated that both medial prefrontal cortex (mPFC) rTMS and right DLPFC cTBS reduced gambling reinforcement in pathological gamblers (24).

A recent pilot study showed that 4 sessions of iTBS added to cognitive-behavioral therapy (CBT) improved nicotine abstinence. The study provided evidence for a potential effect of additional iTBS on nicotine dependence and for lasting effects, enhanced abstinence rates for up to three months (25). To data, no reported studies investigating the efficacy of left DLPFC iTBS in reducing craving in AUD patients.

## Specific Aims

The aim of this double-blind randomized controlled trial is to investigate the efficacy of left DLPFC iTBS in a population of alcohol use disorder patients, compared with sham iTBS. The primary outcome is effect on craving and secondary outcomes are longitudinal outcomes on amount of alcohol and relapse rates.

## Methods/Design

### Study Design

This is a randomized, controlled, double-blind, multicenter study carried out at Shanghai Mental Health Center and other hospitals in China. The patients will be randomly (2:1) assigned to the iTBS group or the control group (sham iTBS). The stimulation target is identical in the left DLPFC. A series of evaluations will be taken before the intervention, after the intervention immediately, and 1 and 3 months after the intervention. All participants provide written, informed consent.

### Ethical Considerations

This study is registered in ClinicalTrials (trial number NCT03932149). The ethics approval is received by Institutional Review Board of Shanghai Mental Health Center (SMHC-IRB) and the number is 2019ky-77. Prior to screening, all participants will have written informed consent.

### Inclusion and Exclusion Criteria

Inclusion criteria are (1) a DSM-5 diagnosis of alcohol use disorder, (2) age between 18 and 65, (3) clinical institute withdrawal assessment of alcohol scale, revised (CIWA-Ar) score of <10, (4) able to complete follow-up visits. Exclusion criteria are (1) severe cognitive impairment, (2) current DSM-5 diagnosis of schizophrenia or another psychotic disorder, (3) severe organic diseases, (4) rTMS contraindications (such as a history of epileptic seizures, metal implants near the head).

### Randomization and Masking

Before recruitment begins at each site, a fixed-size randomization table will be generated using a computer-based algorithm which generates a random replacement block. Staff outside the research team will use the randomization table to insert the participant-specific randomized identification numbers and treatment assignment codes into opaque sealed envelopes. After collecting patient details and baseline assessments, the researchers will assign randomized identification numbers to participants to identify treatment assignments. The staff assessing treatment outcomes are different from the staff administering the TMS, so they are also blinded to treatment conditions. Although the assignments are randomized and participants are blinded to treatment arm, they will be instructed to not discuss their treatment assignments with staff or other participants. After the intervention, the participants will complete a paper and pen assessment wherein individuals state if they think they received real or sham, how confident they are, and to write a brief description evaluating the integrity of the blind. Participants will also complete a paper and pen assessment using a visual analogue scale to assess pain to evaluate the integrity of the sham stimulation.

### Study Process

Patients will be recruited and screened for inclusion and exclusion criteria. If a patient passes the screening, he/she will be recruited in the study and sign the informed consent. All the participants who complete the baseline assessments will be randomly assigned to two groups.

**Figure 1:**
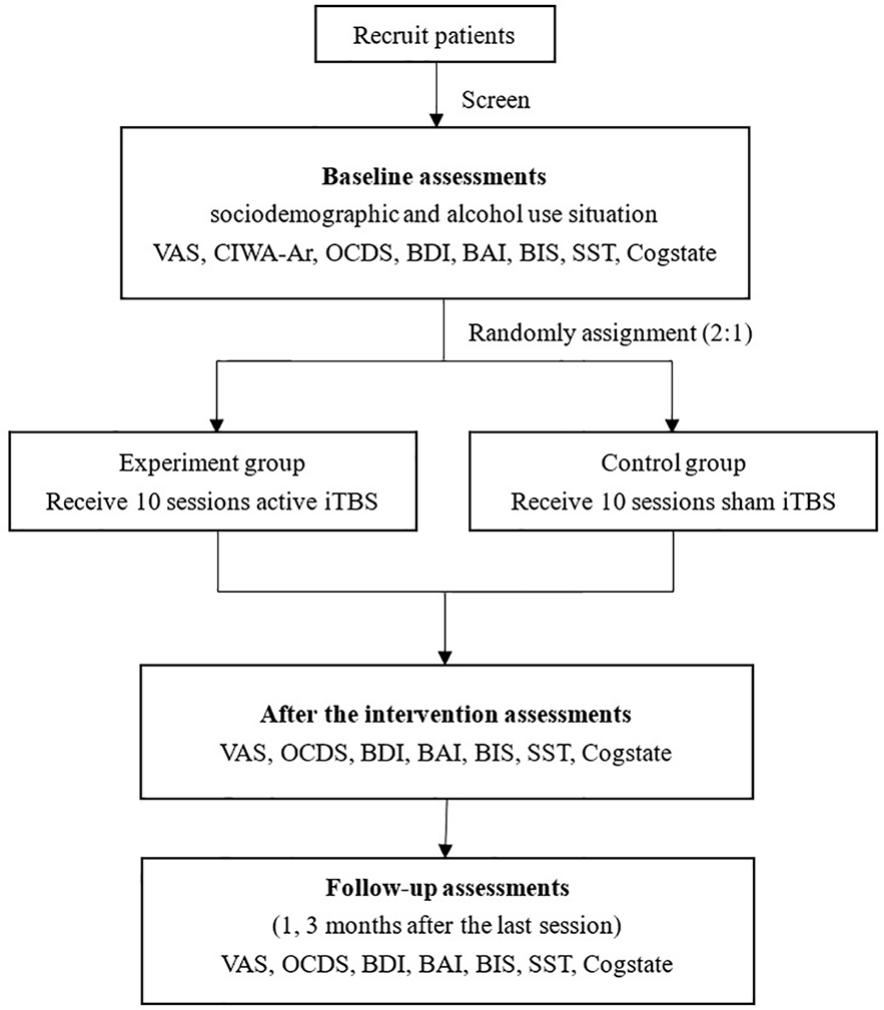
Flow chart of the study.

Both groups will receive 10 sessions (twice daily on weekdays) intervention (experiment group receives active stimulations; control group receives sham stimulations) and finish follow-up assessments after the last session, and three months after the last session. Prior to the study, each participant will be interviewed to assess which type of alcohol they commonly misuse and the glass type they use. Before each session, the participants will be exposed to a variety of alcohol types (wine, liquor) and glass types. They will be able to choose their preferred alcohol variety and touch and/or smell for one minute. A visual analogue scale (VAS) will be used to rate craving. We will use the side effects scale to do the assessment after the intervention. If there are acute and severe side effects, we will stop the intervention, report to IRB office, and provide appropriate treatment to patients based on the contents of inform contents. ”Green channel” for patients has been set up, provide first aid to patient if necessary. The procedure of the study is shown in the ﬂow chart (**Figure 1**).

### Assessments

During the baseline assessments, the sociodemographic and assessment for alcohol use will be documented. Before the intervention, after the last session, and 1 and 3 months after the last session, the following scales are evaluated:

The VAS is a rating scale with few constraints. The VAS consists of a 10-centimeter line on which the respondent marks the degree of their craving. The respondent have great freedom to mark anywhere on the line so that it will show the exact intensity of their craving and their response style (26).

Obsessive compulsive drinking scale (OCDS), a 14-item quick and reliable self-rating instrument provides a total and two subscale scores that measures urge and compulsion aspects of alcohol “craving” (27).

Clinical institute withdrawal assessment of alcohol scale, revised (CIWA-Ar) is a shortened scale with 10 items to measure the severity of alcohol withdrawal syndrome. The shortened version increases efficiency with similar validity and reliability in clinical care and clinical trials on alcohol withdrawal (28).

Beck depression inventory (BDI) is a self-report inventory with 21 items to evaluate the severity of depression (29).

Beck anxiety inventory (BAI) is a self-report inventory with 21 items to evaluate the severity of anxiety (30).

Barratt impulsiveness scale (BIS) is a 30-item self-report scale to measure impulsiveness and it is used widely (31).

In addition to the scales, the following behavioural tasks will be conducted:

Stop signal task (SST) is a behavioral task to measure response inhibition (impulse control). The participant is told to select the left-hand button when they see a left-pointing arrow and select the right-hand button when they see a right-pointing arrow, but if they hear an auditory signal (a beep), they should withhold their response and not select a button. The outcome measure is stop signal reaction time (SSRT).

Cogstate computerized cognitive assessment tool (CCAT), include international shopping list task (ISLT), detection task (DET), identification task (IDN), two back task (TWOB), continuous paired associate learning task (CPAL), groton maze learning test (GML), and social-emotional cognition task (SEC) to evaluate cognitive functions.

### Interventions

The TBS will be administered using a MagVenture MagPro R30 machine, connected to a figure-of-eight-formed 90 mm coil held tangentially to the skull. Each participant's resting motor threshold (RMT) is defined as the minimum intensity that produces minimal motor-evoked potentials (MEPs). It requires peak-to-peak amplitude of about 50-μV in at least five of 10 stimulations. The target is left DLPFC and the iTBS protocol is as follows: 80% of RMT; triplet 50 Hz bursts, repeated at 5 Hz; 2 s on and 8 s off; 600 pulses a session lasting a total of 3 min. The treatment comprises 10 sessions in total, which consist of twice-daily sessions (one time in the morning and one time in the afternoon, 10 sessions a week). The sham stimulations use the same protocol with the coil rotated 180° away from the skull. Patients in sham group could hear the sound of the coil, but the electric field could not induce neuronal activation in their brain.

During the iTBS sessions, the participants will be told to keep their eyes open and to relax. After each session, the participants will report adverse events.

### Primary Outcome Measures

The primary outcome of the study will be the decrease of VAS scores from baseline to the end of treatment.

### Secondary Outcome Measures

The secondary outcome measures of the study will be: the abstinence rate after the treatment, defined as the number of abstinent days in the three months after the last simulation session; the decrease of PSQI, BDI, BAI, BIS scores; the decrease of SSRT; and the increase of Cogstate tasks scores.

### Statistical Analyses

To our knowledge no studies have investigated the effect of left DLPFC iTBS on the craving of alcohol use disorder patients. Therefore, to estimate the sample size we would need, we estimate the sample size referring to a previous study with a similar design, which investigated the efﬁcacy of repetitive transcranial magnetic stimulation in alcohol dependence (18). According to that previous study, we hypothesized that we need at least a similar number of participants (30 participants in the active experiment group). Because we need to conduct three months follow-up assessments, considering a drop-out percentage of 10% (32), the current study will include 38–40 patients in the active stimulation group, resulting in a total of approximately 60 participants.

The data will be analyzed using the Statistical Package for Social Sciences (SPSS). The alpha level will be set at P < 0.05 (two-tailed) for statistical hypothesis. Descriptive analyses will be conducted to determine whether randomization procedure resulted in two groups having no differences in distribution of demographic factors. Appropriate parametric and non-parametric statistical tests will be conducted to analyze descriptive statistics. Multiple comparison corrections will be conducted if necessary. To compare two groups at post-treatment and follow-up assessments, mixed models will be applied and the analysis of covariance (ANCOVA) will be applied to control the eﬀects of covariates (e.g., age, alcohol use history). If the data are not normally distributed, nonparametric tests will be applied.

## Discussion

Currently the treatment of alcohol use disorder is limited to psychosocial and pharmacological treatment. However, these treatments still have greater than 50% relapses of all treated patients within one year suggesting only a moderate effect (33). With the development of rTMS, the treatment of alcohol use disorder has new potential. iTBS is a newer form of rTMS which has been shown to improve the induction of synaptic long-term potentiation (34). The effect is similar to high frequency rTMS but in a shorter time. This paper presents a double blind randomized clinical trial protocol investigating the effect of left DLPFC iTBS on treating alcohol use disorder.

This study requires a three months follow-up assessment, which may be the challenge of the study. It may be difficult for alcohol dependent individuals to complete the whole procedure, so the rate of withdrawal may also higher (35). As we may not obtain a sufficient sample size to adequately assess the longitudinal abstinence rate after the treatment; this outcome measure is the secondary outcome. According to previous studies, we assume the sample size is reasonable. If the dropout rate is too high to meet the requirements of the current study, we will report to IRB and recruit participants according to the protocol requirements.

The limitation of this study is lack of objective measures to assess the alcohol use at the follow up phase, since alcohol metabolizes so fast. In the next step, we hope to find appropriate measures to evaluate the alcohol use accurately.

To our knowledge, this is the first randomized, multicenter trial to investigate the effect of left DLPFC iTBS on treating alcohol use disorder. If we show that left DLPFC iTBS is effective for alcohol use disorder, it may provide a potential treatment which is accessible, well-tolerated, and clinically useful.

### Suspension of Clinical Trial Standards

If the patient is unable to complete the relevant examination, assessment, or cannot complete the follow-up at the specified time within the agreed time, or initiate a suspension study, the clinical trial will be suspended.

### Adverse Event Report

Adverse events that occurred during the study will be notified to the principal investigator as soon as possible, and the primary investigator and the research doctor will jointly determine the treatment plan. Serious adverse events will be reported to the main investigator, ethics committee, and relevant administrative departments within 24 h and processed in a timely manner.

### Data Management and Quality Control

The case report forms completed by the study doctor, the results of the scale assessment and all test results will be kept in the researcher's computer and office in paper or electronic form. This study has a dedicated inspector responsible for the data administrator, and a regular meeting of the research group (once a month) to feed back the research progress and related issues.

All evaluators and data analysts in this study have received relevant professional training to ensure the quality of the enrollment and the quality of post-processing.

## Ethics Statement

The studies involving human participants were reviewed and approved by IRB, Shanghai Mental Health Center. The patients/participants provided their written informed consent to participate in this study.

## Author Contributions

JD and CY conceived and designed the protocol and will lead the study implementation. HS, TC, and VV revised study and assessments methods. All authors revised the protocol critically for important intellectual content.

## Funding

This work was supported by the National Natural Science Foundation Program (81871045) and the Program of Shanghai Science and Technology Committee (19411969200).

## Conflict of Interest

The authors declare that the research was conducted in the absence of any commercial or financial relationships that could be construed as a potential conflict of interest.

## Abbreviations

rTMS, Repetitive transcranial magnetic stimulation; cTBS, Continuous theta burst stimulation; iTBS, Intermittent theta burst stimulation; mPFC, medial prefrontal cortex; DLPFC, dorsolateral prefrontal cortex; DD, delay discounting; VAS, Visual analogue scale; PACS, Penn alcohol craving scale; CIWA-Ar, Clinical institute withdrawal assessment of alcohol scale, revised; BDI, Beck depression inventory; BAI, Beck anxiety inventory; BIS, Barratt impulsiveness scale; SST, Stop signal task; SSRT, Stop signal reaction time; RMT, Resting motor threshold.
